# Etiologies of Persistent Aminotransferase Elevations in Chronic Hepatitis B Patients Treated with Nucleos(t)ide Analogs

**DOI:** 10.5152/tjg.2024.23512

**Published:** 2024-06-01

**Authors:** Qing-Fang Xiong, Lei Zou, Zi-Jie Chen, Hong-Li Liu, Yu-Jia Lu, Du-Xian Liu, Yong-Feng Yang

**Affiliations:** 1 Department of Liver Disease, The Second Hospital of Nanjing, Nanjing University of Chinese Medicine, Nanjing, China; 2 The Clinical Infectious Disease Center of Nanjing, Nanjing, China; 3 Department of Clinical Medicine, Hubei University of Science and Technology, Xianning, China; 4 Southeast University School of Medicine, Nanjing, China; 5 Department of Pathology, The Second Hospital of Nanjing, Nanjing University of Chinese Medicine, Nanjing, China

**Keywords:** Hepatitis B, liver, nucleotide, treatment, transaminase, histopathology

## Abstract

**Background/Aims:**

Recent studies revealed that patients with persistent aminotransferase elevations after antiviral treatment had higher risk of hepatic events; yet its underlying causes remain unclear. Our study aimed to investigate the etiologies of persistent aminotransferase elevations in patients treated with nucleos(t)ide analogs (NAs).

**Materials and Methods:**

A retrospective study was conducted on chronic hepatitis B (CHB) patients who had been receiving NA treatment for over a year and had an aminotransferase level greater than 40 IU/mL (more than twice, with a 3-month interval) and subsequently underwent a liver biopsy.

**Results:**

The study group included 46 patients (34 males) with a mean age of 44.8 ± 20.3 years (range: 24-71 years).The average duration of NA therapy was 3.7 years (1.1-10.6 years). The etiologies of persistant transaminase elevation were categorized into 4 groups: patients with low hepatitis B virus (HBV) viral load (LVL, n = 11); concurrent non-alcoholic fatty liver disease (NAFLD, n = 12); concurrent other liver diseases (OLD, n = 12); and unknown liver dysfunction (ULD, n = 11). The proportion of G ≥ 2 inflammation was significantly higher in the LVL group (90.9%) compared to NAFLD (33.3%), OLD (50%), and ULD (27.2%) groups (*P* = .012). The hepatitis B e-antigen (HBeAg)-positive group exhibited a younger age (34.5 ± 10.2 vs. 48.1 ± 9.4 years, *P* < .001), a lower proportion of fibrosis F ≥ 2 (36.3% vs. 77.1%, *P* = .012), and a higher prevalence of detectable HBV DNA (54.5% vs.14.2%, *P* = .00632) compared to the HBeAg-negative group.

**Conclusion:**

The etiology of persistent aminotransferase elevations in CHB patients undergoing NAs treatment warrants investigation. Besides the commonly observed NAFLD and low HBV viral load, concurrent presence of other liver diseases requires elucidation.The proportion of G≥2 inflammation was higher in the LVL group.

## Introduction

Hepatitis B virus (HBV) infection is a major cause of cirrhosis and hepatocellular carcinoma (HCC), both of which endanger global health and impose a significant economic burden. Long-term administration of nucleos(t)ide analogs (NAs) can inhibit HBV replication, resulting in biochemical remission, histological improvement, and decreasing the incidence of hepatic decompensation and HCC.^1-4^

The biochemical response is one of the important indicators for judging the effect of NA treatment in chronic hepatitis B (CHB) patients. Early alanine aminotransferase (ALT) or aspartate aminotransferase (AST) normalization after NA treatment has been shown to reduce the development of cirrhosis and HCC.^5^ However, even after 1 year of treatment with tenofovir disoproxil fumarate (TDF) or entecavir (ETV), 2 first-line therapies with strong barriers to resistance, the rates of ALT normalization were reported to be 68%-76% and 67%-78%, respectively.^5,6^ There is limited information on the possible causes of the persistent aminotransferase elevations in CHB patients treated with NAs.

Studies have revealed that aminotransferase normalization is influenced by many factors, including hepatitis B e-antigen (HBeAg), HBV DNA level, gender, and the presence of cirrhosis.^3-7^ The etiologies of persistent aminotransferase elevations in CHB patients with complete virus suppression may be associated with other liver injuries: most commonly concurrent alcoholic consumption or non-alcoholic fatty liver disease (NAFLD); co-infection with hepatitis C virus (HCV),^8^ hepatitis D virus (HDV),^9^ hepatitis E virus (HEV), Human Immunodeficiency Virus (HIV), Epstein–Barr virus (EBV), and cytomegalovirus (CMV), and concurrent rare liver diseases such as autoimmune liver disease, metabolic or hereditary liver diseases.^10^ Because these rare liver diseases are challenging to diagnose using standard laboratory tests, liver pathology or even genetic information is frequently required. However, few people had a liver biopsy, which frequently resulted in clinical misdiagnosis. On the other hand, persistently elevated serum aminotransferase levels increase the risk of fulminant hepatitis, cirrhosis, and HCC.^11^ Early detection of the cause and treatment are especially crucial to slow down or prevent the progression of the disease and improve the long-term prognosis of patients.

Herein, we retrospectively analyzed our CHB patients who had been taking NAs for more than a year and had ALT levels higher than 40IU/mL (more than twice, with a 3-month interval) and underwent a liver biopsy to promote early identification of the etiology of abnormal ALT levels and initiate timely treatment.

## Materials and Methods

### Study Population

The study group included CHB patients who underwent liver biopsy at the Second Hospital of Nanjing between January 2018 and December 2020 and met the following criteria: those who had been taking NAs for more than a year and had an ALT level greater than 40 IU/mL (more than twice, with a 3-month interval). Exclusion criteria included co-infection with HCV, HDV, HEV, HIV, EBV, or CMV; alcoholism (20 g/day), history of HCC, current interferon therapy, use of NAs for chemoprevention, and poor treatment compliance of patients. The relevant guidelines were used to make the diagnoses of autoimmune hepatitis (AIH),^12^ primary biliary cholangitis (PBC),^13^ drug-induced liver injury (DILI),^14^ progressive familial intrahepatic cholestasis (PFIC),^15^ hemochromatosis,^16^ idiopathic portal hypertension (IPH), Budd-Chiari syndrome (BCS), and congenital hepatic fibrosis (CHF).^17^ The assessment of medication adherence involved the utilization of a questionnaire in conjunction with the hospital’s medication prescribing records.

This was a retrospective observational study, and the reported data was analyzed anonymously. All participants provided informed consent. The study protocol was approved by the Medical Ethics Committee of the Second Hospital of Nanjing (2018-LY-kt001, date: January 5, 2018).

## Laboratory Methods

A blood routine was performed by the blood cell analyzer of Shenzhen Maiduan Company (BC-3000). The biochemical indexes were detected by OLYMPUS AU2700 automatic biochemical analyzer. Hepatitis B serologic tests were detected by Abbott’s automatic chemiluminescence analyzer. HBV DNA was analyzed by StepOnePlus polymerase chain reaction (PCR) fluorescence analyzer (lower detection limit at 20 IU/mL; Roche Diagnostic System Inc, Branchburg, NJ, USA). Drug-resistant mutations were detected by the American ABI 3730XI sequencer. The serum immunoglobulin and its matching reagents were detected by the Deling BNII plasma protein analyzer (SIEMENS, Germany). Autoimmune antibodies were detected using an indirect immunofluorescence assay in conjunction with Western blot, with kits provided by Germany (EUROIMMUN) Medical Laboratory Diagnostics Co, LTD.

### Liver Biopsy and Histopathology Assessment

All patients had an ultrasound-guided liver biopsy with a 16G needle using the US-made Bard automatic adjustable biopsy device. The liver tissue obtained must have a length greater than 2 cm and include a minimum of 11 portal areas. After fixation, embedding, and continuous sections, hematoxylin and eosin (H&E), and Masson staining of the reticular fiber was performed on liver tissue samples, or special staining was performed under specific conditions. Liver samples were evaluated in a blinded manner by 2 experienced liver pathologists. Liver tissue inflammation (G) and fibrosis (F) were graded according to The METAVIR scoring system, and steatosis was evaluated using the NAS scoring system.^18^ A score of greater than or equal to 5 correlated with the diagnosis of NASH. Likewise, scores less than 3 correlated with ‘‘non-NASH’’ and scores of 3 or 4 were regarded as borderline. When pathological changes from 2 diseases coexisted, abnormal ALT caused by pathological changes was regarded as the final diagnosis.

### Statistical Analysis

The measurement data was expressed as mean ± SD (X ± SD), and the categorical variable data was compared by the chi-squared test or two-tailed Fisher’s exact test. Normally distributed continuous variable data was compared by *t*-test, and non-normally distributed continuous variable data was compared by Mann–Whitney *U*-test as appropriate, and continuous variables by ANOVA. A *P* value of less than 0.05 was considered significant. The results were analyzed using the Statistical Package for Social Sciences (SPSS®) software, version 19.0, for Windows (SPSS Inc., Chicago, Ill, USA).

## Results

### Demographics of Chronic Hepatitis B Patients

Initially, 50 CHB patients were enrolled. Two patients with co-infection HDV, 1 with co-infection HCV and 1 with concurrent alcoholic hepatitis, were excluded, leaving a final enrollment of 46 patients. Among the 46 patients, 34 were males and 12 were females, with an average age of 44.8 ± 20.3 years (range: 24-71 years). Out of the 46 patients, 23.9% (11/46) were HBeAg positivity and 23.9% (11/46) were cirrhotic. Among the cirrhotics, 72.7% (8/11) were classified as child A and 27.2% (3/11) as child B. The average duration of antiviral therapy was 3.7 years (range: 1.1-10.6 years) (Table 1). There were 50% (23/46) patients with G ≥ 2 and 67.3% (31/46) of the patients had F ≥ 2.

There were 23.91% (11/46) of patients with G1 inflammation, 26.08% (12/46) with G1-2, 34.7% (16/46) with G2, 4.3% (2/46) with G2-3, 8.6% (4/46) with G3, and 2.1% (1/46) of patients with G3-4. The fibrosis score was F0-1 in 6.5% (3/46) of the patients, F1 in 17.3% (8/46), F1-2 in 13% (6/46), F2 in 15.2% (7/46), F2-3 in 6.5% (3/46), F3 in 17.3% (8/46), F3-4 in 15.2% (7/46), and F4 in 8.6% (4/46) of the patients.

The NAS scores of 12 NAFLD patients were 3 points in 8.3% (1/12), 4 in 16.6% (2/12), 5 in 33.3% (4/12), 6 in 25% (3/12), and 7 in 16.6% (2/12).

### Etiologies of Persistent Alanine Aminotransferase Elevations

There were 11 patients (23.9%, 11/46) in the low viral load (LVL) group. The HBV DNA loads were between 23.4 IU/mL and 579 IU/mL. Resistance testing revealed lamivudine (LAM) genotype resistance (rtL180M, rtM204V/I, rtV173L) in 3 patients and adefovir Dipivoxil (ADV) genotype resistance (rtA181V/T, rtN236T, rtN238A) in 3 patients. Entecavir (ETV) genotype resistance (rtT184A/I, rtI169T, rtS202G, rtV207I) was observed in 2 patients. No genotype resistance was discovered in the remaining 3 patients with low viral load. After 6 months of TDF or TAF plus ETV combination therapy, all patients achieved aminotransferase normalization and virological response.

**Figure 1.**
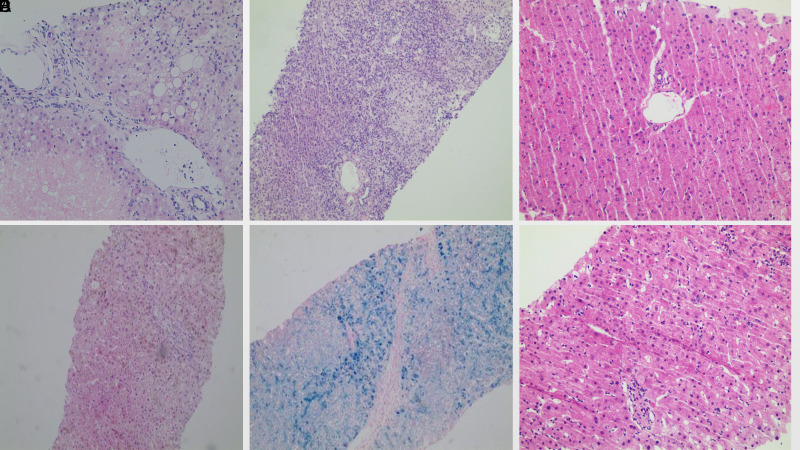
Representative pictures of liver pathology: (A) A 27-year-old male’s liver biopsy displayed fatty degeneration in 30% of the hepatocytes, along with scattered, marked ballooning, and spotty necrosis (H&E 30×). (B) A 54-year-old female exhibited ANA 1 : 320 and IgG 16.8 g/L; liver pathology indicated moderate–severe interface inflammation and plasma cell infiltration (H&E 20×). (C) A 47-year-old male had a dilated portal vein in two-thirds of the portal area, accompanied by a thickened wall, extending into the liver parenchyma (H&E 20×). (D and E) A 50-year-old male had ferritin levels of 2000 ng/mL and transferrin saturation at 95%; liver biopsy showed brown particles in the bile ducts and hepatocytes (D, H&E 20×),confirmed by Prussian staining positive (E, 20×). (F) In a 55-year-old male, liver pathology revealed diffuse focal necrosis in lobules, waxy Kupffer cells in acinus zone 3, and mild interface inflammation (H&E 20×).

**Figure 2.**
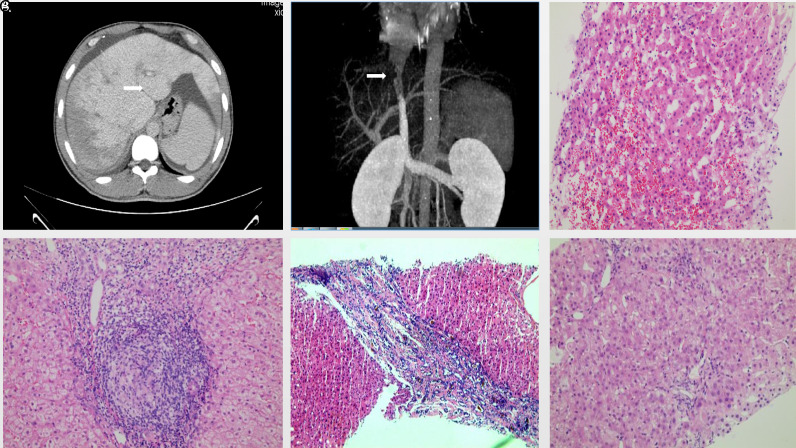
Representative CT and liver pathology images:(A-C) 54-year-old female’s CT scan revealed hydrothorax, ascites, caudate lobe hypertrophy, abnormal hepatic perfusion (A), and hepatic segment inferior vena cava stenosis (B). Liver pathology showed focal hepatic sinus dilatation and congestion in acinus zones 3 (C, H&E 20×). D) A 60-year-old female tested negative for Anti-mitochondrial antibodies, had IgM levels at 4.18 g/L, and exhibited lymphoid follicles, granulomas, and bile duct injury in the portal area (H&E 20×). (E) A 31-year-old male’s liver pathology displayed abnormal changes in the bile duct plate (H&E 20×). (F) A 31-year-old male’s liver biopsy revealed ductopenia in over two-thirds of the portal areas and devoid of lymphoid follicles and granulomas (H&E 20×). CT, computed tomography.

In the NAFLD group, there were 12 patients (26.1%, 12/46), with 9 of them (75%, 9/12) diagnosed with NASH. One patient in this group had a positive HBV DNA level (56 IU/mL), but the NAS score was 6 points (Figure 1A), elevated transaminase was attributed to NAFLD as the primary etiology. There were 12 patients (26.1%, 12/46) in the OLD group. Among them, 2 cases (16.7%, 2/12) for each were identified for AIH (Figure 1B), IPH (Figure 1C), secondary hemochromatosis (Figure 1D and 1E) and DILI (Figure 1F). Additionally, single cases of (8.3%) BCS (Figure 2A - C), PBC (Figure 2D), CHF (Figure 2E), and PFIC type 3 [Figure 2F, ABCB4(7q21|NM_000443.3) Exon15 c.1865G>A p.(Gly622Glu) heterozygous mutation] were detected.

Eleven of the 46 patients (23.91%) were in the ULD group, with 54.54% (6/11) having cirrhosis.

### Comparison of the Clinical and Pathologic Features of Chronic Hepatitis B Patients in the Low Viral Load, Non-alcoholic Fatty Liver Disease, Other Liver Diseases, and Unknown Liver Dysfunction Groups

There were no significant differences in age, gender, body mass index (BMI), white blood cell (WBC), platelet (PLT), ALT, AST, glutamyl transpeptidase (GGT), alkaline phosphatase (ALP), triglyceride, total cholesterol, and proportion of F ≥ 2 among the 4 groups (*P* > .05). The proportion of G ≥ 2 in the LVL group was significantly higher than that in the NAFLD group, OLD group, and ULD group (90.9% vs. 33.3% vs. 50% vs. 27.2%; *χ*^2^ = 10.97, *P* = .012), while there was no significant difference among the other 3 groups (33.3% vs. 50% vs. 27.2%; *χ*^2^ = 1.383, *P* = .50082) (Table 2).

### Clinical and Pathologic Features of HBeAg-Positive and HBeAg-Negative Chronic Hepatitis B Patients

Compared to the HBeAg negative group, the HBeAg positive group had a younger age (34.54 ± 10.26 vs. 48.14 ± 9.4years, *P* < .001), lower proportion of F ≥ 2 (36.3% vs. 77.1%, *χ*^2^ = 6.333, *P* = .012), more detectable HBV DNA (54.5% vs. 14.2%, *χ*^2^ = 7.456, *P* = .00632). No significant differences were observed in terms of gender, WBC, PLT, ALT, AST, GGT, ALP, triglyceride, total cholesterol, and the proportion of G ≥ 2 between the 2 groups (*P* > .05) (Table 3).

## Discussion

According to liver histopathology, the etiology of persistent aminotransferase elevations was low HBV viral load, concurrent NAFLD, other liver diseases, and unknown. The incidence of cirrhosis and HCC was higher in the partial virological response group compared to the virological response group during NA treatment.^4^ Despite the use of NAs with a high barrier to resistance [ETV, TDF, and tenofovir alafenamide (TAF)], some patients exhibited detectable HBV DNA even after several years of treatment, with the detection rates after 1, 2, and 3 years ranging from 7%-33%, 9%-25%, and 10%-17%, respectively.^3,4^ In this study, the average duration of NA treatment was 3.7 years (range: 1.1-10.6 years), with an HBV DNA detection rate of 26.08%, surpassing prior study rates. This discrepancy might stem from earlier use of agents with low resistance barrier (LAM, telbivudine, or ADV),^3,4,19,20^ reducing the efficacy of subsequent ETV or TDF rescue therapy, a notion supported by resistance testing. Additionally, the proportion of G ≥ 2 inflammation was 90.9% in the LVL group. This emphasizes the necessity for adjusting ongoing antiviral treatments and advocates for employing a sensitive PCR assay for HBV DNA detection in CHB patients receiving NAs.

About 54.54% of patients in the ULD group had confirmed liver cirrhosis (F > 3), previous studies had shown liver cirrhosis as a risk factor for poor biochemical response in CHB patients.^3,4,19^ Further research is necessary to ascertain its role in this context. Concurrently, only 27.27% of the patients in this group had G ≥ 2 inflammation. Thus, the treatment remained unchanged and was still in the follow-up stage.

Non-alcoholic fatty liver disease was confirmed in 26.08% of the patients in this study, and it was one of the most common causes of persistent transaminase elevations during NA therapy.^10,21^ The proportion of G ≥ 2 inflammation was observed in 33.3% of the patients, lower than in the LVL group, owing to distinct pathological changes in CHB and NAFLD. CHB typically exhibits portal inflammation, while NAFLD manifests macrovesicular or mixed macrovesicular steatosis, primarily in the acinar zone 3, along with or without hepatocyte ballooning, mixed lobular inflammatory cell infiltration, and perisinusoidal fibrosis.^10^ Studies highlight significant interconnection between the HBV virus molecules and major NAFLD pathways; for instance, liver steatosis may relate to HBV-X protein and farnesoid X receptor.^22^ However, the impact of HBV infection on NAFLD occurrence or vice versa remains uncertain. While steatosis is presumed to accelerate fibrosis progression in chronic hepatitis C, the proportion of fibrosis F ≥ 2 in this group didn’t significantly differ from the other 3 groups, indicating potential unrelatedness between fibrosis and fatty degeneration. Concurrently, diet control and exercise intervention were implemented alongside NA treatment.

In this study, 2 CHB patients with concurrent AIH were distinguished by the presence of moderate-to-severe interface hepatitis, portal plasma cell infiltrate, and hepatocyte rosette formation, emperipolesis; whereas CHB presented a mild interface hepatitis.^12^ The International Autoimmune Hepatitis Group diagnostic scoring system was used to confirm AIH.^8,12,23^ Two patients received immunosuppressive treatment with prednisolone while undergoing NA therapy, successfully achieving remission. Drug-induced liver injury is distinguished by explicit medication and supplement exposure, necrosis of centrilobular zone 3, and the absence of severe fibrosis or cirrhosis.^24^ Notably, there were no flares in liver function following glucocorticoid withdrawal.

Iron deposition was identified in 2 cases via liver biopsy, indicated by Prussian blue staining levels of 3+ and 4+, separately. In CHB patients, iron metabolism disorders are associated with HBV infection. Serum iron overload can exacerbate liver iron deposition, inflammation, and progress liver fibrosis, leading to ALT elevations.^25^ Treatment included the administration of iron chelators alongside intermittent intravenous phlebotomies. One patient showed gradual improvement, while the other patient couldn’t tolerate iron chelators and underwent a liver transplant in 2019.

IPH exhibited distinct hepatic lobular architecture, multiple dilated portal veins with thickened walls, penetrating the liver parenchyma, and the absence of fibrosis.^17^ The patient diagnosed with BCS presented hepatic segment inferior vena cava stenosis on CT scan and exhibited focal hepatic sinus dilatation and congestion within acinus zone 3 in the liver biopsy. She underwent a transjugular intrahepatic portosystemic shunt and was promptly administered indefinite anticoagulant medication in order to reduce the risk of clot extension and new thrombotic episodes.^17^

In this study, a PBC patient was Anti-mitochondrial antibodies(AMA)negative, posing challenges for clinical diagnosis. Another PFIC3 patient exhibited ductopenia in the liver biopsy specimen and the ABCB4[(7q21|NM_000443.3) Exon15 c.1865G>A p.(Gly622Glu)] heterozygous mutation. One patient with CHF presented typical bile duct plate malformation pathology.^26,27^ they received treatment for cholestasis with ursodeoxycholic acid (10-15 mg/kg/day), resulting in normal transaminase levels during the follow-up period. This subset of patients proves challenging to diagnose solely based on clinical and immunological findings, underscoring the necessity of liver histopathological analysis for accurate diagnosis.

The HBeAg-negative CHB patients exhibited a significantly older age, a higher proportion of fibrosis F ≥ 2, and a lower prevalence of detectable HBV DNA compared to the HBeAg-positive patients. It is possible that HBeAg-negative CHB develops primarily from HBeAg-positive cases, which has a longer disease duration and long-term immune damage. The heightened prevalence of detectable HBV DNA in HBeAg-positive CHB patients may be related to their inadequate virological response due to initially higher viral load at baseline.^28-30^

Data derived from real-life cohorts represent a patient spectrum broader than that found in randomized controlled trials, which frequently exclude individuals with multiple comorbidities. Consequently, these results hold greater relevance for routine clinical practice. Nevertheless, our study has a few limitations. First, only a subset of patients with persistently elevated ALT during antiviral therapy underwent liver biopsy. Secondly, the study was constrained by a small sample size.

In conclusion, patients with persistent aminotransferase elevations after NA therapy require immunological, histopathological, and even molecular genetic tests. These evaluations aim to identify potential concurrent liver diseases. Additionally, detection of HBV DNA using sensitive PCR testing is essential to exclude the possibility of low viremia.

## Figures and Tables

**Table 1.** Baseline Characteristics of Chronic Hepatitis B Patients

Parameters	mean ± SD or % (n)
Age (years)	44.89 ± 20.32
Gender, male (%, n)	73.9(34)
PLT (×10^9^/L)	128.89 ± 40.78
TB (μmol/L)	18.98 ± 7.21
ALT (IU/L)	73.08 ± 24.12
AST (IU/L)	50.47 ± 37.45
HBeAg positivity (%, n)	26.08 (12)
Concurrent diabetes (%, n)	13.04 (6)
BMI (kg/m^2^)	23.5 ± 7.12
Cirrhosis (%, n)	23.91(11)
ETV/TDF/Others (%, n)	45.6/39.1/15.2 (21/18/7)

ALT, alanine aminotransferase; AST, aspartate aminotransferase; BMI, body mass index; ETV, entecavir; PLT, platelet; TB, total bilirubin; TDF, tenofovir disoproxil fumarate.

**Table 2.** Comparison of Clinical and Pathological Features of CHB Patients in the Low Viral Load, Non-alcoholic Fatty Liver Disease, Other Liver Diseases, and Unknown Liver Dysfunction Groups

Parameters	LVL (n = 11)	NAFLD (n = 12)	OLD (n = 12)	ULD (n = 11)	Statistic Value	*P*
Gender, male (%, n)	81.8 (9)	66.6 (8)	75 (9)	72.7 (8)	0.699	.874
BMI (kg/m^2^)	22.36 ± 1.62	23.83 ± 4.13	24.33 ± 2.90	23.00 ± 2.56	0.986	.408
Age (years)	41.63 ± 12.14	48.00 ± 10.96	46.16 ± 9.22	43.36 ± 13.47	0.701	.557
WBC (×10^9^/L)	5.09 ± 1.24	5.71 ± 1.96	5.41 ± 1.76	5.21 ± 1.74	0.243	.865
PLT (×10^9^/L)	130.44 ± 58.20	144.72 ± 48.57	119.54 ± 67.47	116.71 ± 64.44	0.447	.721
ALT (IU/L)	73.02 ± 25.92	107.05 ± 53.42	51.67 ± 32.66	55.87 ± 20.38	1.553	.218
AST (IU/L)	48.29 ± 35.62	58.84 ± 39.31	57.10 ± 32.85	32.35 ± 30.79	1.064	.337
GGT (U/L), median (range)	143 (12.0-209)	175 (21.0-245.0)	169.0 (19.9-1752)	112.5 (21.3-270)	3.089	.378
ALP (IU/L)	53.31 ± 36.85	42.67 ± 30.76	136.99 ± 105.25	81.11 ± 79.77	1.379	.265
Triglyceride (mmol/L), median (range)	1.1 (0.31-3.87)	1.35 (0.52-17.7)	1.165 (0.81-1.75)	0.9 (0.65-2.2)	1.576	.665
Total cholesterol (mmol/L)	3.76 ± 0.86	4.78 ± 1.61	3.91 ± 1.07	3.64 ± 1.34	1.961	.135
G ≥ 2 (%,n)	90.9 (10)	33.3 (4)	50 (6)	27.2 (3)	10.97	.012
F ≥ 2 (%,n)	81.8 (9)	58.3 (7)	75 (9)	54.5 (6)	2.632	.452

ALP, alkaline phosphatase; ALT, alanine aminotransferase; AST, aspartate aminotransferase; BMI, body mass index; GGT, glutamyl transpeptidase; LVL, low viral load; NAFLD, non-alcoholic fatty liver disease; OLD, other liver diseases; PLT, platelets; ULD, unknown liver dysfunction; WBC, white blood cells.

**Table 3.** Clinical and Pathological Features Between HBeAg-Positive and HBeAg-Negative Patients

Parameters	HBeAg^+^ (n = 11)	HBeAg^−^ (n = 35)	Statistic Value	*P*
Gender, male (%, n)	81.8 (9)	71.4% (25)	0.469	.4934
Age (years)	34.54 ± 10.26	48.14 ± 9.74	3.989	.000
WBC (×10^9^/L)	5.49 ± 1.31	5.34 ± 1.79	0.241	.811
PLT (×10^9^/L)	145.80 ± 52.67	122.85 ± 59.95	1.070	.292
ALT (IU/L)	50.85 ± 27.16	80.74 ± 75.09	1.223	.229
AST (IU/L)	40.57 ± 30.26	53.89 ± 36.54	1.035	.307
GGT (U/L), median (range)	137.5 (12-1752)	172 (21-397)	1.576	.115
ALP (IU/L), median (range)	64.6 (15.8-738)	45.3 (9.4-250.3)	0.289	.772
Triglyceride (mmol/L)	1.30 ± 0.54	1.70 ± 2.87	0.456	.650
Total cholesterol (mmol/L)	3.69 ± 0.92	4.14 ± 1.39	1.013	.317
G ≥ 2 (%, n)	45.4 (5)	51.4 (18)	0.119	.7301
F ≥ 2 (%, n)	36.3 (4)	77.1 (27)	6.333	.012
Detectable HBV DNA (%, n)	54.5 (6)	14.2 (5)	7.456	.00632

ALT, alanine aminotransferase; ALP, alkaline phosphatase; AST: aspartate aminotransferase; GGT: glutamyl transpeptidase; HBeAg, hepatitis B e-antigen; PLT, platelets; WBC, white blood cells.
